# Lung function, asthma symptoms, and quality of life for children in public housing in Boston: a case-series analysis

**DOI:** 10.1186/1476-069X-3-13

**Published:** 2004-12-07

**Authors:** Jonathan I Levy, LK Welker-Hood, Jane E Clougherty, Robin E Dodson, Suzanne Steinbach, HP Hynes

**Affiliations:** 1 Harvard School of Public Health, Department of Environmental Health, Landmark Center Room 404K, P.O. Box 15677, Boston, MA, 02215, USA; 2 School of Nursing, University of Texas Medical Branch, 301 University Boulevard, Galveston, TX, 77555-1029, USA; 3 Department of Pediatrics, Boston Medical Center, 715 Albany St., Boston, MA, 02118, USA; 4 Boston University School of Public Health, Department of Environmental Health, 715 Albany St., Boston, MA, 02118, USA

## Abstract

**Background:**

Children in urban public housing are at high risk for asthma, given elevated environmental and social exposures and suboptimal medical care. For a multifactorial disease like asthma, design of intervention studies can be influenced by the relative prevalence of key risk factors. To better understand risk factors for asthma morbidity in the context of an environmental intervention study, we conducted a detailed baseline evaluation of 78 children (aged 4–17 years) from three public housing developments in Boston.

**Methods:**

Asthmatic children and their caregivers were recruited between April 2002 and January 2003. We conducted intake interviews that captured a detailed family and medical history, including questions regarding asthma symptom severity, access to health care, medication usage, and psychological stress. Quality of life was evaluated for both the child and caregiver with an asthma-specific scale. Pulmonary function was measured with a portable spirometer, and allergy testing for common indoor and outdoor allergens was conducted with skin testing using the prick puncture method. Exploratory linear and logistic regression models evaluating predictors of respiratory symptoms, quality of life, and pulmonary function were conducted using SAS.

**Results:**

We found high rates of obesity (56%) and allergies to indoor contaminants such as cockroaches (59%) and dust mites (59%). Only 36% of children with persistent asthma reported being prescribed any daily controller medication, and most did not have an asthma action plan or a peak flow meter. One-time lung function measures were poorly correlated with respiratory symptoms or quality of life, which were significantly correlated with each other. In multivariate regression models, household size, body mass index, and environmental tobacco smoke exposure were positively associated with respiratory symptom severity (p < 0.10). Symptom severity was negatively associated with asthma-related quality of life for the child and the caregiver, with caregiver (but not child) quality of life significantly influenced by caregiver stress and whether the child was in the intensive care unit at birth.

**Conclusion:**

Given the elevated prevalence of multiple risk factors, coordinated improvements in the social environment, the built environment, and in medical management would likely yield the greatest health benefits in this high-risk population.

## Background

Asthma morbidity and mortality have been increasing in recent years, with a disproportionate impact on urban minority children [1-4]. Hospitalization and morbidity rates have been shown to be elevated for nonwhites versus whites [3] and in inner-city settings with low-income populations [4]. Multiple recent studies have attempted to explain these disparities by evaluating environmental exposures and housing conditions, racial/ethnic variations, poverty, and social or psychological factors, with no definitive conclusions regarding the dominant factors [1,2,5-9].

Regardless of the relative contributions of these and other factors, children in urban public housing are important to consider, because they likely have elevated exposures across numerous domains, some of which could be addressed through development-wide interventions. However, there has been only limited evaluation to date of asthma in this high-risk subpopulation [10,11].

The Healthy Public Housing Initiative (HPHI) is a collaborative effort that includes the Boston Housing Authority (BHA), West Broadway and Franklin Hill Tenant Task Forces, Committee for Boston Public Housing, Boston Public Health Commission, Boston University and Harvard University Schools of Public Health, and Tufts University School of Medicine. A primary goal of HPHI is to evaluate the effectiveness of interventions in reducing known asthma triggers and improving the health of pediatric asthmatics in public housing in Boston.

The effectiveness of environmental interventions in this context will clearly depend on the prevalence of environmentally-linked risk factors within this cohort (i.e., allergy status), as well as the prevalence of other risk factors for asthma morbidity. In addition, when evaluating the efficacy of environmental interventions, numerous health endpoints may be valuable to consider. Health care utilization will inform cost-effectiveness analyses, typically driven by infrequent but severe events such as hospitalizations [12,13]. On the other hand, self-rated quality of life can capture a broad array of activity-based and psychosocial outcomes, and pulmonary function measures or respiratory symptoms provide more objective and sensitive markers of health improvements.

Past studies have demonstrated varied relationships among these parameters. For example, percent of predicted forced expiratory volume in one second (FEV1%) has been correlated with asthma attacks [14] and symptom score but not with symptom days [15]. Asthma-related quality of life was correlated with FEV1% in a low-income adult population [16] but not in a general population sample [17]. FEV1% was correlated with asthma-related quality of life in mild asthmatics, but was a weaker predictor than symptom intensity and was not correlated significantly for more severe asthmatics [18]. Finally, pediatric asthma symptoms have been correlated with asthma-related quality of life but not FEV1% or measures of asthma control, with relationships that vary by age of the child [19]. However, none of these studies focused on low-income pediatric populations.

More broadly, past studies have generally not considered the full array of risk factors and health endpoints for inner-city asthmatics. The most comprehensive assessment to date has been the National Cooperative Inner-City Asthma Study (NCICAS), which evaluated many similar endpoints as our study in a longitudinal baseline assessment [20], although self-rated quality of life was not considered in this publication. In order to understand the characteristics of asthmatic children in our longitudinal intervention study and to determine the relationships among key health endpoints, we conducted an extensive baseline assessment for all children enrolled in our study.

Thus, the objective of our analysis is to characterize the baseline risk factors and health status of a cohort of asthmatic children enrolled in an intervention study based in public housing developments in Boston and to determine concordance between and risk factors for key health endpoints (e.g., pulmonary function, respiratory symptoms, and self-reported quality of life). We hypothesize that risk factors associated with housing quality and psychosocial stress will be elevated in our cohort when compared with reference groups, and that quality of life will be significantly influenced by asthma symptom severity and other caregiver characteristics.

## Methods

We recruited asthmatic children from the Franklin Hill, West Broadway, and Washington Beech public housing developments in Boston (located in the neighborhoods of Dorchester, South Boston, and Roslindale, respectively). Recruitment was coordinated by Community Health Advocates, residents of the developments or surrounding neighborhoods who were involved in outreach and data collection, following training about asthma, its risk factors, and interviewing techniques. Recruitment methods included advertised enrollment open houses, community meetings, mailbox drops for flyer circulation, and door knocking. Any children between the ages of 4 and 17, who lived in the developments, had self-reported doctor-diagnosed asthma, and who were willing to enroll in a longitudinal intervention study, were eligible. Enrollment occurred between April 2002 and January 2003. Written informed consent was obtained from all caregivers, with assent forms completed by children above the age of 8, and the protocols were approved by the institutional review boards of all three participating universities.

In the intake interview, the caregiver (defined as the individual who knows most about asthma care for the child) was asked about family demographics, child and family asthma history, access to health care, exposure to smoking, and medication usage, with questions taken from NCICAS when possible to facilitate comparability [20]. Because psychological stress has been shown elsewhere to be a strong predictor of immune function [21] and airway inflammation and obstruction [22], the caregiver was given the Cohen four-item abbreviated Perceived Stress Scale [23]. In addition, she was asked about neighborhood social cohesion and exposure to violence [24], factors that have been related to respiratory symptoms and other measures of asthma morbidity [25]. Finally, she was surveyed about the influence her child's asthma had on her quality of life, using the Paediatric Asthma Caregiver's Quality of Life Questionnaire (PACQLQ) [26].

The Paediatric Asthma Quality of Life Questionnaire (PAQLQ) [27] was used to determine the influence of asthma on the child's quality of life, administered to the caregiver for children age 7 and younger and directly to children age 8 and older. In addition, we evaluated quality of life using the EuroQol EQ5D self-report questionnaire [28] combined with previously published formulas [29], providing a comparison between an asthma-specific scale and a general health status scale. The EQ5D also included a visual analogue scale (VAS).

Pulmonary function was assessed using the NDD EasyOne Diagnostic portable spirometer (NDD Medical Technologies, Andover, MA), an instrument which correlates well with office-based spirometry [30]. Although pulmonary function was measured longitudinally within the intervention study, we focus on the baseline assessment (conducted concurrently with the intake interview). To compare lung function across children, we determined the percent of predicted value for FEV1 and peak expiratory flow (PEF) using standard reference equations [31]. Spirometry results and questions regarding symptom severity and medication use were used to classify asthma severity following NHLBI guidelines [32]. In addition, given reported height and weight, we calculated body mass index (BMI) and used age-specific BMI distributions [33] to categorize children as overweight (above 95^th ^percentile), at risk of overweight (85^th ^to 95^th ^percentile), or not at risk (below 85^th ^percentile).

Allergy testing was conducted using similar methods as NCICAS [20], with skin testing using the prick puncture method. Valid tests had a negative control wheal at least 1 mm smaller than the positive histamine wheal, and tests were considered positive if the wheal for a given allergen exceeded the negative control wheal by at least 2 mm. Allergens evaluated included an 11-tree mix, a 7-grass mix, ragweed, dog, cat, mouse, cockroach, *D. pteronyssinus*, *D. farinae*, *Alternaria*, *Aspergillus fumigatus*, *Cladosporium*, and *Penicillium*.

For our exploratory regression models, we evaluated the relationship between our primary outcome measures (FEV1 % predicted, a respiratory symptom score, and caregiver and child asthma-related quality of life) and a subset of demographic variables, intrinsic risk factors, health care risk factors, physical risk factors, and social risk factors. Furthermore, we considered FEV1% as a potential predictor of the respiratory symptom score, and both FEV1% and the respiratory symptom score as hypothetical predictors of quality of life. We treated FEV1% in a logistic regression, using 80% of predicted FEV1 as the cutoff for low FEV1%, and evaluated other health outcomes in linear regressions. Given missing data and numerous covariates, we conducted an initial screen using univariate regressions (retaining variables for which p < 0.2), and then constructed a multivariate stepwise regression with p < 0.1 as the entry and exit criteria. Finally, for covariates with extensive missing data, we constructed multivariate regressions both with and without these terms to evaluate the sensitivity of our findings. All statistical analyses were conducted using SAS version 8.02, using PROC REG for linear regressions and PROC LOGISTIC for logistic regressions.

## Results

### Demographics and risk factors

In total, 78 children from 61 households were enrolled in the HPHI intervention study. As indicated in Table 1, 41 (53%) of these children were from Franklin Hill, with 27 (35%) from West Broadway and 10 (13%) from Washington Beech. The mean age at the time of enrollment was 8.7 years (median = 8.0), with a similar age distribution across the three developments. A majority of participants (64%) self-reported as Hispanic, with 33% self-reporting as black or African-American.

**Table 1:** Baseline demographic characteristics of asthmatic children in three public housing developments in Boston

	Franklin Hill	West Broadway	Washington Beech	Total
Number of children	41	27	10	78
Age (%)				
< 6	27%	30%	40%	29%
6–9	32%	26%	20%	28%
10–12	22%	30%	20%	24%
>= 13	20%	15%	20%	18%
Race/Ethnicity (%) *				
Hispanic	61%	67%	70%	64%
African-American	41%	22%	30%	33%
Caucasian	0%	11%	0%	4%

* Race/ethnicity was asked in an open-ended question, so respondents could indicate both Hispanic status and race. So, the total can be greater than 100%.

Considering prominent non-environmental risk factors associated with asthma, 16% of children were in an intensive care unit upon birth and 10% were on a respirator. Seventy percent of children had a parent or grandparent with asthma, while 43% had eczema or hay fever and 34% had a family history of eczema or hay fever. For the 75 children with recorded height and weight, 56% were categorized as overweight and 9% were categorized as at risk of overweight. Forty-two percent of children lived with a smoker, and 45% of children were around smokers at least several times per month.

**Figure 1:**
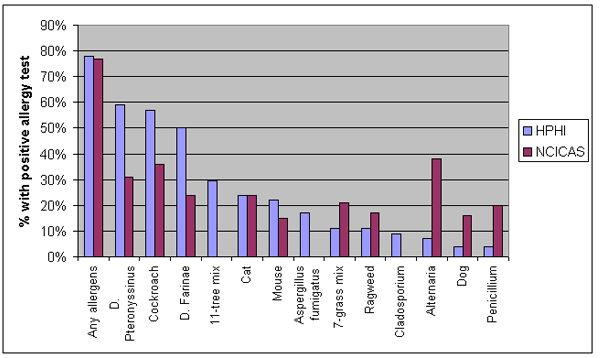
Prevalence of allergies among asthmatic children in HPHI and NCICAS [20].

For the subset of 46 children (59%) who underwent allergy testing, 78% were sensitized to at least one of the tested substances, with the most prevalent allergies including *D. pteronyssinu*s (59%), cockroach (59%), *D. farinae *(50%), and tree pollen (30%) (Figure 1). Only 44% of these allergic children were reported to have allergies by their caregivers.

### Medical care

For the 70 children for whom we received information about medications, 87% were taking short-acting beta-agonists at the time of the survey. Thirty-one percent of these children on beta-agonists also reported the use of any long-term asthma control medication (corticosteroids, leukotriene modifiers, or mast cell stabilizers). All children who did not report the use of beta-agonists indicated that they were using long-term control medication. Seventeen percent of children indicated that they were currently taking allergy medication as part of their asthma control. Of note, for the remaining eight children, we could not ascertain whether non-responses indicated lack of any medication or lack of recall/medication availability.

There were additional factors and barriers that indicated potentially sub-optimal asthma care (Table 2). Although most (92%) children had current health insurance coverage that paid some portion of asthma-related medical expenses (with 97% covered by Medicaid/MassHealth during the past year), for 28% of children, the caregiver reported having no doctor to call other than the emergency room for asthma care. Only 37% of children had a written asthma action plan signed by their doctors. Fifty-four percent of children had a spacer to use with their inhalers. Furthermore, only 27% of children had a peak flow meter, with only 19% ever using it at home or school. Children with peak flow meters tended to be slightly older than those without peak flow meters, with a similar distribution of severity. As indicated in Table 2, the asthma management practices often differed significantly across developments.

**Table 2:** Access to medical care and asthma management practices for children in three public housing developments in Boston

	Franklin Hill	West Broadway	Washington Beech	Total	p-value (Wilcoxon rank-sum test)
% with doctor to call other than emergency room	68% (N = 41)	89% (N = 27)	29% (N = 7)	72% (N = 75)	0.005
% with written asthma action plan	39% (N = 41)	46% (N = 24)	10% (N = 10)	37% (N = 75)	0.14
% with peak flow meter	28% (N = 40)	33% (N = 27)	10% (N = 10)	27%	0.37
% of persistent asthmatics using long-term control medication	21% (N = 19)	57% (N = 21)	14% (N = 7)	36% (N = 47)	0.03

In addition, 71% of caregivers indicated that at least one of seven barriers impeded asthma management for their children during the last six months. The most frequently cited barrier was that the pharmacy did not have their asthma medication (38%), followed by the asthmatic child either not being home when it was time to take the medicine (29%) or refusing to take the medicine (26%). The cost of the medication was cited as a barrier by only 12% of caregivers, the lowest percentage among the seven questions, an indication that MassHealth/Medicaid coverage was largely viewed as adequate.

### Social stressors

**Figure 2:**
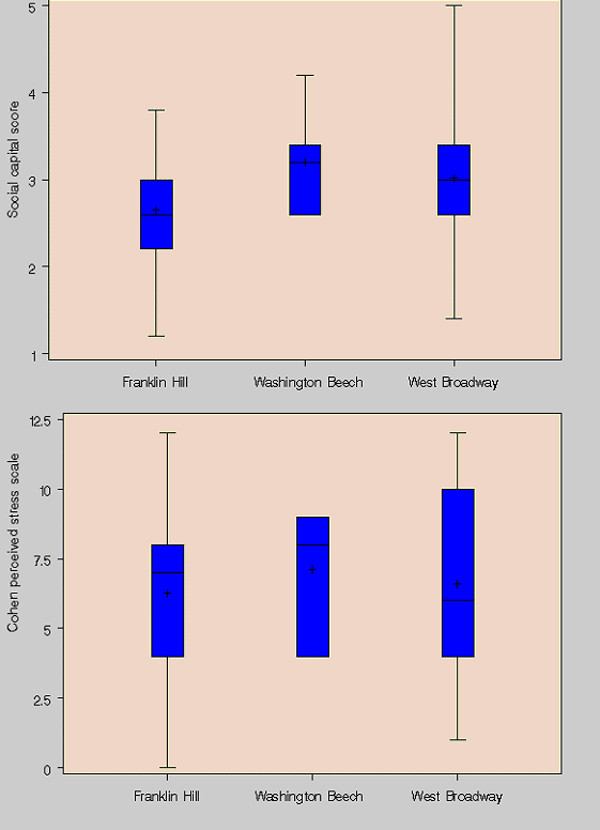
**Social cohesion and perceived stress for caregivers of asthmatic children in HPHI. **Social cohesion scores range from 1–5 (5 = maximum, 1 = minimum), while Cohen perceived stress scale scores range from 0–16 (16 = maximum, 0 = minimum).

On the five-point social cohesion scale, there was a significant difference across developments, with a lower mean value at Franklin Hill than at West Broadway or Washington Beech (Figure 2). There was, however, much greater variability in perceived social cohesion within rather than across developments, consistent with previous findings [24].

We also found significant differences across developments in exposure to violence. We asked caregivers whether they were afraid that they or their children would be hurt by violence in their neighborhood, whether they have had violence used against them or other household members in their neighborhood, and whether they fear letting their children play outside in their neighborhood because of community violence (Table 3). In all cases, the highest rates were reported at Franklin Hill. At Franklin Hill, but not at the other developments, the responses to these questions depended on the age of the asthmatic child. For example, 84% of caregivers of asthmatic children under 8 reported fear of violence, versus 29% of caregivers of asthmatic children 8 and older (with 54% of caregivers of children under 8 reporting having violence used against their household, versus 13% for caregivers of older children). In spite of these facts, there was no significant difference in the Cohen Perceived Stress Scale between developments, with more substantial within-development variability (Figure 2).

**Table 3:** Exposure to violence for caregivers of asthmatic children in three public housing developments in Boston

	Franklin Hill	West Broadway	Washington Beech	Total	p-value (Wilcoxon rank-sum test)
% afraid of violence in neighborhood	63% (N = 30)	20% (N = 20)	43% (N = 7)	46% (N = 57)	0.01
% directly impacted by violence in neighborhood	41% (N = 32)	14% (N = 22)	0% (N = 7)	26% (N = 59)	0.02
% not let children play outside due to violence in neighborhood	60% (N = 30)	23% (N = 20)	14% (N = 7)	41% (N = 59)	0.009

### Asthma severity and symptoms

As indicated in Table 4, a majority of children reported having wheezing or tightness in their chest, needing to slow down or stop their activities due to their asthma, or having nighttime asthma symptoms within the last two weeks, an indication of poorly controlled asthma. In addition, six percent of the children were reported to have a severe asthma attack (unable to say more than one or two words between breaths) in the last two weeks. Three of the children (4%) reported staying overnight in the hospital for asthma during the last two months. Although a small fraction of the cohort, this would correspond (if sustained) to an annual asthma hospitalization rate of 23% in this population.

**Table 4:** Frequency of reported asthma symptoms within two weeks prior to enrollment in intervention study

	Never	1–2 times/week	3–6 times/week	At least daily
Wheezing, tightness in the chest, or cough (N = 74)	20%	41%	24%	15%
Slow down/stop play or activities (N = 74)	34%	35%	19%	12%
	
	Never	1–2 times	3–4 times	At least 5 times
	
Wake up at night (N = 76)	32%	34%	25%	9%

### Spirometry

For the subset of 49 children age six or older able to perform spirometry, the mean FEV1% was 88% (median of 88%, standard deviation of 15%). Twenty-nine percent of children had FEV1 less than 80% of predicted, though no values were less than 60% of predicted. The mean PEF% was 97% (median of 96%, standard deviation of 17%). Twelve percent of children had PEF less than 80% of predicted, with none having PEF less than 60% of predicted. As would be anticipated, these two measures were well correlated (Spearman correlation of 0.69), with all but one of the children with PEF below 80% of predicted also having FEV1 below 80% of predicted.

Of note, of the children under 6 tested, seven (30%) were able to record acceptable spirometry values. Only a child who had been recently hospitalized for asthma exacerbation had reduced FEV1 and PEF, both below 60% of predicted [31]. Given poor performance and issues in selecting appropriate reference equations for young children [34], spirometry for children under the age of 6 is not considered further in our analysis.

Based on the four questions addressing recent asthma symptoms (as summarized in Table 4), spirometry measures, and medications prescribed and used, we determined that a majority of the children in our cohort (56% of those with complete information) would be considered to have moderate persistent asthma, with 14% considered severe persistent, 10% mild persistent, and 20% mild intermittent. Of note, of the 47 children categorized as having persistent asthma who provided information on their medications, only 36% reported being prescribed any daily controller medication (63% of severe persistent, 33% of moderate persistent, and 17% of mild persistent children).

### Quality of life

**Figure 3:**
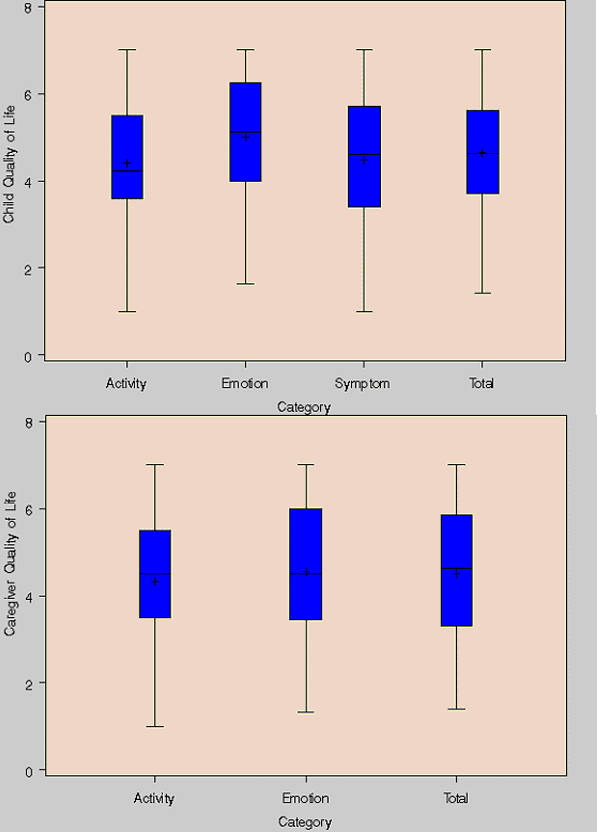
**Distribution of asthma-related quality of life scores for asthmatic children and their caregivers. **Asthma-related quality of life scores range from 1–7 (7 = maximum, 1 = minimum).

For children, using the PAQLQ, the median score on the seven-point respiratory symptoms subscale was 4.6, with medians of 4.2 for activity limitation, and 5.1 for emotional function, with a median overall quality of life score of 4.6 (where a score of 1 indicates maximal impairment and a score of 7 indicates no impairment). The range across children was substantial, with overall quality of life scores ranging from 1.4 to 7 (Figure 3). Similarly, caregivers reported median PACQLQ scores of 4.3 for activity limitation and 4.6 for emotional function, with an overall median score of 4.5 but a range from 1.4 to 7. Using the EQ5D, the median health-related quality of life was 0.81 (range from 0.28 to 1), while the visual analogue scale (VAS) yields a median quality of life score of 80 (range from 30 to 100). The wide ranges indicate some of the limitations in interpreting these values cross-sectionally rather than longitudinally.

The quality of life questionnaires provide insight regarding the perceived burdens of asthma beyond the aggregate quality of life scores. For example, in the EQ5D responses, 31% of children were reported to be moderately or extremely worried or depressed, with 50% of children having problems doing their usual activities and 53% of children in moderate or extreme pain or discomfort. In addition, 39% of caregivers reported that their child's asthma interfered with their job or work around the house at least some of the time, with 62% reporting sleepless nights related to their child's asthma at least some of the time.

### Correlation and regression analyses

One key question is whether any of the primary measures of asthma severity (lung function, quality of life, and respiratory symptoms) are significantly correlated with one another in a cross-sectional baseline assessment. For lung function, we consider both FEV1% and PEF%. Quality of life outcomes include the aggregate PAQLQ score for the child, aggregate EQ5D and VAS scores for the child, and the aggregate PACQLQ scores for the caregiver. For respiratory symptoms, we develop a symptom score reflecting the responses to the questions in Table 4 as well as having a severe asthma attack in the last two weeks. The symptom score ranges from zero to eight, with a maximum of two points assigned for each question and a higher score reflecting more frequent symptoms. While this scale is simple and implicitly places equal weight on respiratory outcomes of differing severity, it is similar to scales developed elsewhere and reasonably captures the gradient in symptom frequency and severity.

Considering the Spearman correlations among these key covariates (Table 5), the lung function measures are strongly correlated with one another but are not significantly correlated with either the respiratory symptom score or quality of life. In contrast, the respiratory symptom score is significantly correlated with the VAS and both child and caregiver asthma-related quality of life, with lower quality of life given higher symptom frequency as anticipated. The various quality of life scales are generally significantly correlated with one another, with slightly weaker relationships for the EQ5D scale, which is not specific to asthma. Not surprisingly, the correlation between caregiver and child asthma-related quality of life is stronger for children age 7 and under, where the caregiver evaluates the child's quality of life (r = 0.75), than for children age 8 and older, where the child evaluates their own quality of life (r = 0.31).

**Table 5:** Spearman correlation coefficients between respiratory symptom score, quality of life measures, and lung function

	Symptom score	EQ5D	VAS	Child AQL	Caregiver AQL	FEV1%
EQ5D	-0.07	-	-	-	-	-
VAS	-0.29 *	0.20	-	-	-	-
Child AQL	-0.43 **	0.44 **	0.43 **	-	-	-
Caregiver AQL	-0.46 **	0.27 *	0.28 *	0.49 **	-	-
FEV1%	-0.12	0.14	-0.24	-0.08	-0.07	-
PEF%	-0.03	0.05	-0.24	-0.09	0.08	0.65 **

*: p < 0.05, **: p < 0.01

Given our sample size, missing data for selected covariates (such as spirometry or allergy status), and the number of factors hypothesized to influence our outcome measures, we consider our regression analyses to be exploratory in nature. The goal is to better understand the above correlations and factors that might influence the relationships among our outcome measures. For each of the outcome measures, we evaluate a subset of demographic variables (age, race/ethnicity, gender, household size, housing development), intrinsic risk factors (BMI, being in the intensive care unit at birth, eczema), health care risk factors (having a doctor to call other than the emergency room), physical risk factors (allergies to roaches, dust mites, or any agents; or environmental tobacco smoke exposure), and social risk factors (social capital, perceived stress, fear of violence in the neighborhood, and not letting children play outside due to fear of violence in the neighborhood). It should be noted that some of these covariates may be direct causative agents, while others represent proxies or outcomes that could be influenced by asthma severity (such as caregiver stress).

The results from this analysis are summarized in Table 6, including significant terms from univariate and multivariate regressions. Although multiple risk factors were predictive of low FEV1% in univariate regressions (including age, BMI, cockroach allergy, environmental tobacco smoke exposure, and social capital), no terms were statistically significant in multivariate models. The respiratory symptom score was elevated for a variety of physical and social risk factors, with household size, BMI, and environmental tobacco smoke exposure remaining significant in multivariate models. Fewer factors were predictive of child asthma-related quality of life, but the respiratory symptom score was strongly and negatively associated with the PAQLQ score. Similarly, the respiratory symptom score strongly predicted caregiver asthma-related quality of life in both univariate and multivariate models, with a significant influence in multivariate models for the caregiver's perceived stress and for whether the child required NICU care at birth.

**Table 6:** Univariate/multivariate regressions of FEV1%, respiratory symptoms, and quality of life measures on selected risk factors

	FEV% < 80%	Respiratory symptom score	Child asthma-related quality of life	Caregiver asthma-related quality of life
Age	*0.05 (+)*	NS	NS	NS
Hispanic	NS	NS	NS	NS
African-American	NS	NS	NS	NS
Gender	NS	NS	NS	NS
Household size	NS	**0.0007 (+)** **0.009 (+)**	NS	*0.04 (-)*
Housing development	NS	NS	NS	NS
BMI	*0.12 (+)*	**0.03 (+)** **0.02 (+)**	NS	NS
Born in NICU	NS	NS	NS	**0.09 (-)** **0.03 (-)**
Eczema	NS	*0.13 (+)*	NS	NS
Doctor to call other than ER	NS	NS	NS	NS
Allergy to roaches	*0.18 (+)*	NS	NS	*0.19 (+)*
Allergy to dust mites	NS	NS	NS	NS
Any allergies	NS	NS	NS	NS
Environmental tobacco smoke exposure	*0.19 (-)*	**0.03 (+)** **0.08 (+)**	NS	NS
Social capital	*0.15 (+)*	NS	NS	NS
Perceived stress	NS	*0.03 (+)*	*0.04 (-)*	**0.001 (-)** **0.004 (-)**
Fear of violence in neighborhood	NS	*0.06 (+)*	NS	*0.01 (-)*
Not letting children play outside due to fear of violence in neighborhood	NS	*0.10 (+)*	NS	NS
Low FEV%		*0.15 (+)*	**0.13 (+)** **0.04 (+)**	NS
Respiratory symptom score			**0.0002 (-)** **0.02 (-)**	**< 0.0001 (-)** **0.009 (-)**

NS: No statistical significance in univariate regression (p > 0.2)

*Value in italics*: Statistically significant in univariate regression (p < 0.2), but not in multivariate regression. The value presented is the p-value for the univariate regression, and the +/- sign indicates the direction of the relationship

**Values in bold**: Statistically significant in multivariate regression (p < 0.1). The first value presented is the p-value for the univariate regression, and the second value presented is the p-value for the multivariate regression. The +/- sign indicates the direction of the relationship.

## Discussion

To address our first hypothesis and interpret this baseline characterization of asthmatic children in public housing enrolled in an intervention study, it is instructive to compare the prevalence of selected risk factors with those reported in previous studies [20,25,35-39] (Table 7). Within our study, there appear to be a greater percentage of overweight children and children with a family history of asthma, as compared with NCICAS, other studies of low-income asthmatics, and general population studies. The fraction of children with cockroach or dust mite allergies is also high, although *Alternaria *allergy prevalence is quite low, indicating that the most effective interventions might differ between HPHI and NCICAS. In addition, exposure to violence and fear of violence is slightly higher than reported in NCICAS. Although the fraction of children with persistent asthma who are adequately medicated is quite low (36%) and indicative of poorly managed asthma, this figure is actually higher than the percentages reported in other studies, indicating that this is a pervasive problem in asthma management.

**Table 7:** Comparison of children in HPHI intervention study with children in other studies

	HPHI	NCICAS [20]	Other low-income asthmatic populations	Population-based studies
Family history of asthma	70%	57%	-	N/A
% overweight	56%	19%	-	15% [39]
% of families with at least one smoker	39%	59%	46% [35]	41% [36]
% with cockroach allergy	59%	36%	52–78% [1]	22% [36]
% with European dust mite allergy	59%	31%	-	27% [36]
% with *Alternaria *allergy	7%	38%	-	16% [36]
% not let children play outside due to violence in neighborhood	41%	34% [25]	-	-
% of persistent asthmatics on long-term control medication	36%	24% ^1^	27% [37]	26% [38] ^2^

^1 ^All asthmatics

^2 ^Moderate/severe asthmatics only

More broadly, our findings regarding medication usage coupled with the prevalence of children with mild, moderate, or severe persistent asthma and the frequency of respiratory symptoms suggest that this population is being treated sub-optimally. NHLBI guidelines [32] indicate that all individuals with persistent asthma should be prescribed an inhaled steroid or other long-term control medication in addition to a fast-acting beta-agonist. In addition, for moderate or severe persistent asthmatics, it is recommended that long-acting bronchodilators be added to the medication regimen. Compounding the problem of inadequate medication is the relatively low usage of asthma action plans or peak flow meters, which are both part of recommended patient education and self-management activities. These shortfalls could be related to inadequate quality of care, limitations in access to and continuity of care, communication gulfs between caregivers and providers, or other factors, and further investigation is needed to determine the root causes of this management gap.

Although exploratory in nature, our correlation and regression analyses provide some useful insights regarding the factors associated with various measures of asthma severity in our cohort. FEV1% was not strongly correlated with other health measures, and was only weakly associated with a subset of risk factors in univariate regressions. As this measurement was taken at a single point in time at varying times of day and seasons, a weak relationship is unsurprising, especially when compared with symptoms over a two week period [18]. The lack of a relationship may also be related to a relatively small sample size and a small number of participants with low FEV1%. In addition, many asthmatics may be poor perceivers of symptoms of respiratory difficulty, potentially explaining the disconnect between pulmonary function measures and asthma symptoms or quality of life.

The respiratory symptom score was moderately associated with a number of risk factors in univariate regressions, with all coefficients in the anticipated directions (i.e., greater symptom frequency and severity with larger household sizes, higher BMI, reported eczema, higher environmental tobacco smoke exposure, higher psychosocial stress, higher fear of violence, and higher prevalence of low FEV1%).

The robust multivariate relationship with household size and presence of smokers could be indicative of the influence of indoor air quality or respiratory infection related to unit crowding, with the association between obesity and asthma symptom frequency and severity in agreement with past studies [40,41]. The extremely strong relationship between respiratory symptom score and asthma-related quality of life for the child is logical and provides some indication that both measures were reasonably constructed. The relationship between perceived stress and the caregiver's asthma-related quality of life, in addition to the child's respiratory symptoms, may indicate that the acute stresses associated with lack of control over one's surroundings could adversely affect quality of life. Since the NICU term is associated with caregiver's quality of life (but not the child's quality of life), it is less likely a surrogate of early respiratory injury and could be more broadly related to chronic stress about a child's health status. It is also interesting to note that variables such as race, ethnicity, gender, and housing development were not significant predictors in any models.

There are some clear limitations in interpreting the results of our investigation. First, given the numerous risk factors for asthma, both the univariate and multivariate regressions must be interpreted with caution. For example, fear of violence in the neighborhood was significantly associated with reductions in caregiver quality of life in univariate regressions (p = 0.01), but given a strong positive association between fear of violence and caregiver stress as well as the child's respiratory symptom score, this term did not enter into the final regression model. Thus, both the significant and insignificant terms from our regression models should be interpreted with caution, as they may not reflect causal relationships.

In addition, the participants in our study represented a convenience sample of individuals from three selected public housing developments in Boston, who were interested in enrolling in a longitudinal intervention study. It is unclear whether our participants are representative of asthmatic children across those developments, or more broadly, asthmatic children in public housing. Families who enrolled in our study may have been more desperate for help given current asthma conditions, or may have had greater confidence in the ability of a research project to improve their child's health. A comparison between the demographics of our study population and the demographics of the developments indicates that the age distribution and racial/ethnic composition are similar, but there is no way of knowing if our cohort represents typical characteristics of all asthmatic children across the BHA.

In addition, we have evaluated the prevalence of risk factors and correlations among key indicators of respiratory health in a cross-sectional survey without a defined control group. Thus, while we have found an elevated prevalence of obesity and allergies to cockroach and dust mites when compared with other populations, we cannot infer a causal relationship with asthma. However, this comparison illustrates the relative importance of various risk factors internal to our intervention study. In addition, the correlations among respiratory health measures will not necessarily be identical cross-sectionally and longitudinally. For an intervention study, the crucial question is whether observed changes in lung function would occur simultaneously with changes in quality of life or respiratory symptoms, and knowledge of the cross-sectional correlations is not necessarily informative. That being said, the correlations among health endpoints (Table 5) and the regression results (Table 6) do provide some indication that children with more frequent respiratory symptoms have lower quality of life at baseline, making these children candidates for improvements in both health endpoints.

In spite of these limitations, our study provides some important and unique information. To our knowledge, this is the most comprehensive evaluation focused on asthmatic children in public housing to date. Although many attributes of public housing are similar to low-income private housing, the viability of large-scale interventions in public housing based on common indoor environmental exposures and centralized management makes it important to characterize similarities and differences from other low-income populations. More broadly, our study demonstrates that a community-based participatory research paradigm, with members of the community conducting most of the primary data collection, is able to gather valid and meaningful data. Given that recruitment and retention of our study population would have been quite difficult without involvement from community members, it is clear that community-based participatory research is essential for detailed evaluations of asthma in public housing.

The findings from this cross-sectional baseline evaluation of asthmatic children in public housing in Boston have multiple policy implications. The inadequacy of medical care for a majority of asthmatic children indicates that medical interventions might yield substantial improvements in asthma status for poor children. The fact that significant differences existed in adequacy of medical care for asthma across housing developments indicates likely variability across providers (although the data indicate that this problem exists across community health centers and academic medical centers). In addition, the high prevalence of cockroach and dust mite allergies indicates that interventions aimed at reducing or eliminating these triggers are likely to provide health improvements. This reinforces the expectation that interventions in public housing, a setting with high asthma prevalence and high prevalence of allergic responses to indoor contaminants, are likely to be meaningful and effective. Finally, the responses to the psychosocial questions as well as the percentage of caregivers who feared neighborhood violence and modified their children's activities as a result indicates that violence and stress are substantial risk factors for both respiratory health and other outcomes. Our findings point to the need for coordinated improvements in the social environment, the built environment, and in medical management.

Future investigations should similarly evaluate asthma risk factors and severity in other public housing settings to determine whether our conclusions provide generalizable and relevant information for regional or national housing authorities in considering intervention strategies. In addition, a longitudinal comparison of correlations among measures of asthma severity would help determine whether conclusions drawn from a cross-sectional evaluation are robust.

## Conclusions

We conclude that asthmatic children enrolled in a public housing-based intervention study would likely benefit from a coordinated intervention focused on reduction of indoor allergens (especially cockroach and dust mites), improved medical management, and increased social support.

## List of Abbreviations

AQL – Asthma-related quality of life

BHA – Boston Housing Authority

BMI – Body mass index

FEV1 – Forced expiratory volume in one second

FEV1% – Percent of predicted forced expiratory volume in one second

HPHI – Healthy Public Housing Initiative

NCICAS – National Cooperative Inner-City Asthma Study

NHLBI – National Heart, Lung, and Blood Institute

NICU – Neonatal intensive care unit

PACQLQ – Paediatric Asthma Caregiver's Quality of Life Questionnaire

PAQLQ – Paediatric Asthma Quality of Life Questionnaire

PEF – Peak expiratory flow

PEF% – Percent of predicted peak expiratory flow

VAS – Visual analogue scale on EuroQol EQ5D quality of life questionnaire

## Competing interests

The authors declare that they have no competing interests.

## Authors' contributions

JIL participated in study design and implementation, conducted statistical analyses, and drafted the manuscript. LKWH designed and administered survey instruments, provided support to participants as a nurse/case manager, and contributed to analyses related to adequacy of health care. JEC contributed to survey design and analyses for psychosocial risk factors. RED completed a literature review, drafted text, and conducted statistical analyses. SS provided asthma severity classifications and related analyses and conducted allergy testing. HPH collaborated in the conception and design of the study, and co-directed data collection and study coordination. All authors read and approved the final manuscript.
